# Synecology of *Lagoecia cuminoides* L. in Italy and evaluation of functional compounds presence in its water or hydroalcoholic extracts

**DOI:** 10.1038/s41598-023-48065-w

**Published:** 2023-11-27

**Authors:** Enrico V. Perrino, Zuhair N. A. Mahmoud, Francesca Valerio, Valeria Tomaselli, Robert P. Wagensommer, Antonio Trani

**Affiliations:** 1 CIHEAM, Mediterranean Agronomic Institute of Bari, Via Ceglie 9, 70010 Valenzano (Bari), Italy; 2 Institute of Sciences of Food Production, National Research Council of Italy, Via Amendola 122/O, 70126 Bari, Italy; 3 Department of Biosciences, Biotechnologies and Environment, University of Bari “Aldo Moro”, Via Orabona 4, 70125 Bari, Italy; 4 Faculty of Education, Free University of Bozen-Bolzano, Viale Ratisbona 16, 39042 Bressanone (Bolzano), Italy

**Keywords:** Biodiversity, Community ecology, Conservation biology, Biochemistry, Ecology, Plant sciences

## Abstract

*Lagoecia cuminoides* L. is a very rare and threatened taxon in Italy, never studied before for its ecology and potential use for human consumption. Furthermore, few data are available on the biological activities of its metabolites. A phytosociological study was carried out in the only two Italian sites, and its state of conservation was also evaluated according to the IUCN (International Union for Conservation of Nature) protocol. The collected plant material was used to make two types of extracts: hot water infusion to evaluate the use of this plant as tea and hydroalcoholic extraction to evaluate the use of it in herbal liqueur preparation. The presence of functional compounds in the extracts were investigated by gas and liquid chromatography coupled to mass spectrometry techniques. Ten non volatiles compounds were identified in the extracts, most of which derivatives of quercetin. Thirty-five volatiles compounds were also identified in the plant aerial part and extracts belonging to the chemical class of terpenoids, and among them β-farnesene, thymol, γ-terpinene and p-cymene were the most abundant. The species is characterized by compounds known for their health effects and for its potential applications for human consumption, being this species already used as decoction in some countries of Middle East. Thanks to its characteristic behaviour to grow in limiting pedoclimatic conditions this species can be potentially used in organic farms situated in rural marginal areas.

## Introduction

There is an increasing public concern about the harmful effects of chemicals used in conventional agriculture and food production on human health leading to an increasing demand for more natural and healthier food. This habit encourages researchers to investigate about organic grown plants, especially wild species as new source for food or food additives and/or ingredients^1, 2^.

The integration of wild species into crop fields, particularly in organic farms, is of great importance to support the biodiversity and stability of agro-ecology systems and to enhance farmers’ livelihood^3^. This kind of wild species is useful for several reasons, such as the use of their extracts as source of natural herbicidal compounds^4^, used in the food sector, and to increase the plant and animal biodiversity. Some of the wild species recently “domesticated” are aromatic and medicinal plants, which showed economical and agronomical potentials greater than the parental cultivated species^5, 6^.

**Figure 1:**
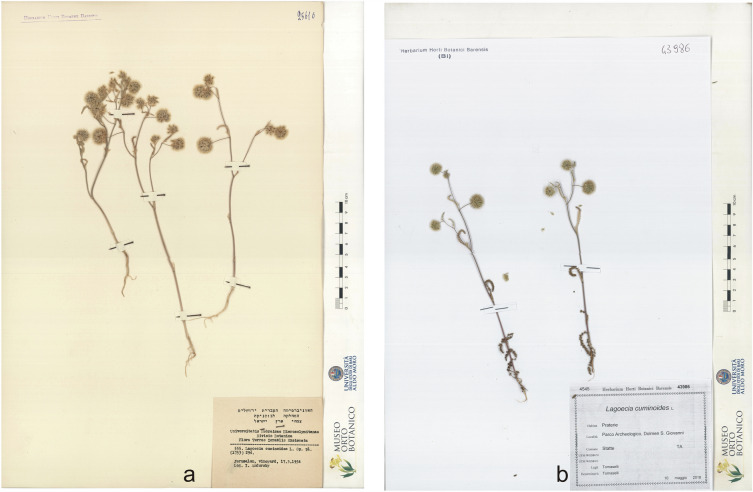
Herbarium samples: (**a**) Jerusalem (Palestine) (BI 25616); (**b**) Statte (Taranto—Italy) (BI 43986).

*Lagoecia cuminoides* L. (Apiaceae), described by Linnaeus^7^, is a Mediterranean-Turanian element, though disjointed being reported as native in Bulgaria, Greece, Crete with Karpathos, East Aegean islands, Cyprus, Israel and Palestine (Fig. 1a), Jordan, Lebanon, Syria, Libya, Portugal, Spain^8^, Albania^9, 10^, North Macedonia^11^, Crimea^12^, Iran^13, 14^, and Iraq^15, 16^. The presence of *L. cuminoides* was considered doubtful in Italy as it had not been reported for a long time from 1925 to 2018 (Fig. 1b), while it is considered an “alien” species, with status as casual, in France, Germany and Norway^17^. In Italy it was probably cultivated since the eighteenth century^18, 19^, for sure growing wild in southern Italy^20–22^, where it was discovered in Apulia, in the province of Taranto at Leucaspide, by D. Profeta, who described its small fruits and its cumin like taste^23^, and confirmed for the same region by other botanists^22, 24^, but after 1912 nobody else recorded it neither in Leucaspide nor in other places of Apulia.

**Figure 2:**
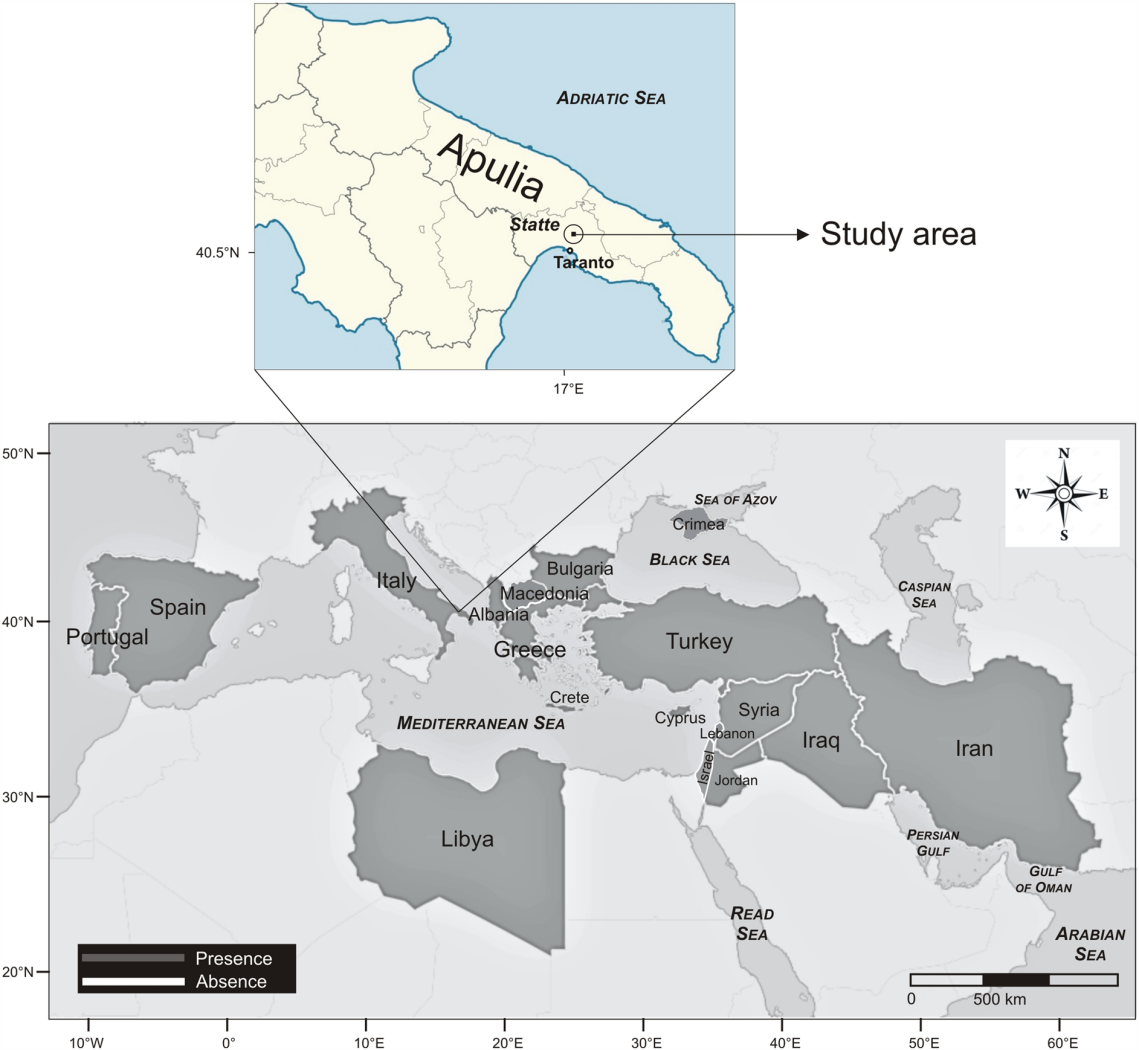
Geographic distribution of *Lagoecia cuminoides* L. in the native countries (updated) and study area (created with CoreIDRAW version 12.0.0.458, https://www.coreldraw.com/).

Thanks to Lattanzi^25^, this taxon was discovered on 2018 in two sites in the municipality of Statte (Taranto province, Apulia), by the Working Group for floristic investigation of the Italian Botanical Society (SBI), and later by other botanists^26^. These Italian reports, together with those of the other Mediterranean countries, allowed to update the distribution map of the species (Fig. 2).

*L. cuminoides* was preserved and cultivated as an aromatic and medicinal plant in various Italian botanical gardens, and Mattioli^27^ gave also information on its use for the treatment of various diseases.

Metabolomics studies on this species and investigation about the functional properties of its extracts, such as antioxidant and antimicrobial activities, have been carried out mainly in Turkey and Iran^14, 28^, whereas no studies exist describing the ecology and the metabolomics of this species in Italy.

The seeds of *L. cuminoides* are used as a cumin substitute, giving to food spicy and aromatic characteristics^29^. It is used importantly in curries as an ingredient, and often is used as a flavour for cakes, bread and biscuits, where it has also a beneficial effect by improving the digestion process^30, 31^. When fully ripe the seeds are harvested and then dried and stored in jars^14^. The aerial part of the plant is frequently used to prepare an infusion for treating gastrointestinal diseases^32^. An essential oil from the seeds is used as a food flavouring^33^.

The main objective of the present work is to investigate the ecological context in which *L. cuminoides* grows in Italy, assess its conservation status in Italy, identify and characterize the metabolic pattern of the plant extract, evaluate their biological activity and finally its potential use in the food sector to establish a link with organic farms.

## History, taxonomy, nomenclature and morphology

The name *Lagoecia* (from the Greek lagṓs oikos: hare’s home) alludes to the inflorescences of the species that resemble the bed made by the hare for her cubs^34^. The name *cuminoides* (from the Greek εἷδος eídos: similar to cumin) linked only to the fragrance of the small seeds which looks like that of *Cuminum cyminum* L. (cumin), native species of Afghanistan, Iran and Iraq^35^, that has been in use as a spice for thousands of years. *C. cyminum* itself is sometimes confused with caraway (*Carum carvi* L.), another spice of the same family (Apiaceae), and often in many European countries it is not clearly distinguished from these latter two species. In addition, Slavic and Uralic languages refer to cumin as "Roman caraway" or "spice caraway". Finally, *Bunium persicum* (Boiss.) B.Fedtsch., *Bunium bulbocastanum* L. and the unrelated *Nigella sativa* L. are sometimes called "black cumin", with the latter belonging to a different family (Ranunculaceae).

### Taxonomy

The taxonomy of *Lagoecia* L. is controversial. The genus was described by Linnaeus^7^, under *Monogynia* (ovary with only one carpel) and not *Digynia* (ovary with two carpels) in which many of Apiaceae species were placed, due to reduction to only one carpel in *Lagoecia* genus^36^. Several botanists suggest a different history from the other Apiaceae. Drude^37^ and Wolff^38^ recognized the tribe *Lagoecieae* with three genera: *Lagoecia*, *Petagnia* Guss., and *Arctopus* L. Calestani^39^ divided Umbelliferae (= Apiaceae) into four subdivisions with the *Lagoecineae* that included the single tribe *Lagoecieae*. Cerceau-Larrival^40^ considered *Lagoecia* a monotypic tribe in *Endressioideae*. Later, Valiejo-Roman^36^ based on genetic studies (sequencing of nuclear ribosomal DNA) showed the affinity of *Lagoecia* with *Crithmum* L., *Trachyspermum* Link, *Scaligeria* DC., *Bunium* L., *Elaeosticta* Fenzl, *Pyramidopter*a Boiss. and *Oedibasis* Koso-Pol., with an outside position from all other *Saniculoideae*. Finally, Doğru-Koca et al.^41^ from a phylogenetic point of view suggested that *Froriepia* K.Koch should be the sister genus of *Lagoecia cuminoides*, and although both genera belong to the same tribe (*Piramidoptereae*), they are morphologically very different from each other. Even if the collocation of the genus *Lagoecia* is complex, from a morpho-taxonomic point of view *Lagoecia* should be considered very close to *Petagnia*, having in common a specific character: the abortion of an ovarian lodge^21^ and the consequent maturation of a single achene.

**Chromosome number**. *Lagoecia cuminoides*: 2n = 16^42^

### Nomenclature

Apiales Nakai (1930)

Apiaceae Lindl. (1836)

*Lagoecia* L. (1753)^7^

*Lagoecia cuminoides* L. (1753)^7^

### Synonyms

*Cuminoides obliqua* Moench in Methodus: 94 (1794); *Cuminum cuminodes* (L.) Kuntze in Revis. Gen. Pl. 1: 266 (1891).

### Morphology

**Figure 3:**
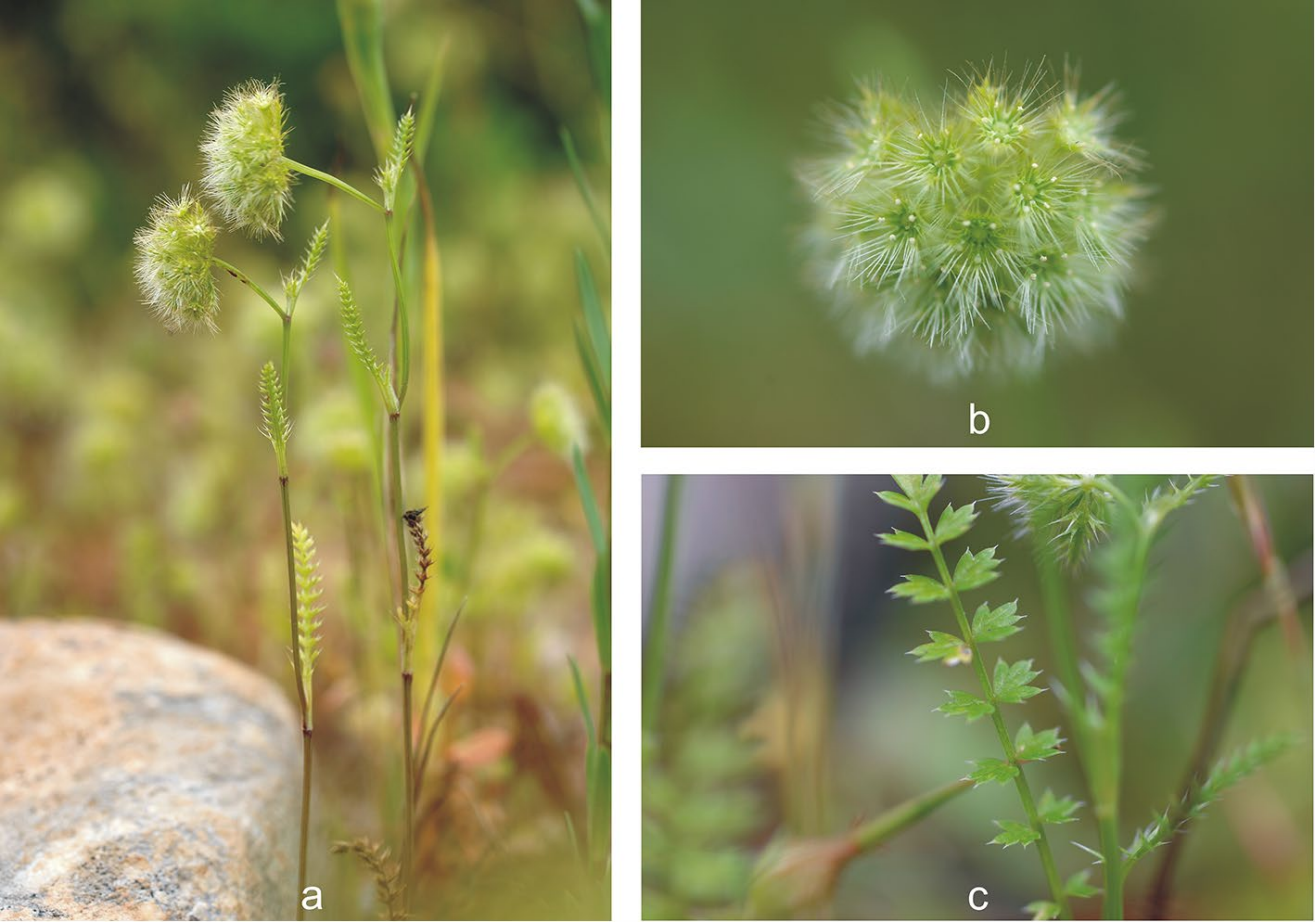
*L. cuminoides*. Habit (**a**), flowers (**b**), and leaf (**c**) at Statte (Taranto), 13 May 2023. Pictures by E.V. Perrino.

*L. cuminoides* is an annual herb, 10–30 cm high if it grows in desired moist soil (Fig. 3). Basal leaves with ovate and dentate segments, those cauline with segments deeply divided into short, lanceolate and aristate lobes. Umbels compound, subspherical, and feathery 0.5–1.5 cm in diameter, dense, globose; rays numerous. Bracts and sepals like leaves. Bracteoles 4, 2-pinnatisect, with setaceous lobes. Sepals pinnatisect, with lobes setaceous and sometimes 2–3-fid. White petals extended into two linear horns. Style 1. Fruit 2 mm, cylindrical-curved, covered with short, brittle, clavate hairs that look-like a pappus^43, 44^.

## Materials and methods

The in-field surveys on the only two known Italian populations (Fig. 1) were conducted in 2022, from April to July. During the surveys some specimens were collected and used for laboratory analysis accordingly to the methods described in the following paragraphs.

### Ecological characterization

Ecological characterization was performed through the phytosociological method of the Zurich–Montpellier school^45^ with information on physiographic data, as relevé identification code, geographic coordinates in WGS84, altitude (m a.s.l.), aspect, slope, relevé area (m^2^), stoniness, rockiness, cover total, average height of herbaceous layer (cm), number of individuals in the population (estimate), and habitat of Directive 92/43/ECC (https://environment.ec.europa.eu/). For the identification of taxa, Flora Europaea^43^ and Flora d’Italia^44^ were used; for nomenclature Bartolucci et al.^46^ and Galasso et al.^47^ were followed and for syntaxonomic nomenclature Mucina et al.^48^ was consulted. The collected plant material was stored at the Herbarium Horti Botanici Barensis of the University of Bari (BI) (3 March 2022, E.V. Perrino).

### Assessment of the conservation status in Italy

The conservation status of the species in Italy was assessed according to the IUCN protocol^49^. The area of occupancy (AOO) was calculated with a 2** × **2 km cell grid, while the extent of occurrence (EOO) was calculated as convex hull.

### Metabolites extraction

Plant samples collected during botanical surveys were air dried at room temperature and in the dark. All aerial parts were used (leaves, stem and flowers). The drying phase was considered accomplished when a constant weight was reached. After the drying phase, the plant material was grinded using a coffee grinder for 15 s. The obtained powder was weighted in amber glass bottles and added of hot water or hydroalcoholic solution using 1:30 w/vol ratio. The water decoction was performed using distilled water heated at 90 °C and 15 min time infusion. Then the extracts were cooled down and filtered using 0.45 µm cellulose recycled filters and stored at − 20 °C until the analysis. For hydroalcoholic extraction, dried vegetable material was added of 70% vol/vol ethanol, and, after mixing, the bottle was kept in the dark for 3 days and mixed every 12 h. The extract was then filtered using 0.45 µm cellulose recycled filters and stored at − 20 °C until the analysis.

### Total polyphenol content

Total polyphenols were determined by spectrophotometric Folin assay according to Wrolstad et al.^50^. In plastic cuvettes 4 mL capacity, were placed 1.58 mL of water HPLC grade, 20 µL of extract and 100 µL of Folin reagent. The cuvettes were covered with parafilm and mixed and left for 5 min timed, then added of 300 µL of freshly prepared Na_2_CO_3_ 20% and mixed. After 90 min the absorbance was read at 765 nm against a blank made at the same way of sample but using clean extraction solution (water or hydroalcoholic solution) instead of sample extract. Calibration was done using gallic acid standard in the range 10–800 mg/L. Results were expressed as mg of gallic acid equivalent on 100 g of plant material dry weight or per mL of extract.

### Total antioxidant activity

Total antioxidant activity was determined using the 2,2′-azino-bis(3-ethylbenzothiazoline-6-sulfonic acid (ABTS) assay calibrated with Trolox. The ABTS radical was obtained by mixing 10 mL of ABTS 7 mM with an equal volume of persulphate 4.95 mM. The mixture was left at room temperature in the dark for 12 h, then stored refrigerated for a maxim of 7 days. Using the stock solution of ABTS radical, a dilution 1:25 was prepared, obtaining an absorbance at λ 730 nm of 0.7 units. The calibration range was 25–800 nmol/mL of Trolox equivalent. The assay was carried out as follow. In plastic cuvettes of 1 cm of optical length, 980 µL of ABTS diluted radical solution were placed and then 20 µL of sample or standard added. The cuvettes were closed using parafilm, mixed and left for 25 min timed. A blank was also prepared in the same manner but using the extraction solution instead of sample. After 25 min the absorbance was read at 730 nm against a cuvette with water. The difference between the sample and the blank containing only the extraction solution was determined and used in the calculation and expressed as µmol/kg of Trolox Equivalent Antioxidant Capacity (TEAC).

### Non-volatiles metabolites identification by liquid chromatography coupled with mass spectrometry (LC/MS)

Three microliters of the extract were injected in the UHPLC Ultimate 3000 system (Dionex Thermo Fisher Scientific) equipped with LPG-3400RS pump, WPS-3000 autosampler, TCC-3000 column oven, and a Photodiode Array Detector PDA 3000. Chromatographic separation was obtained by the column Zorbax Eclipse XDB C_18_, 10 cm of length, 2.1 mm of internal diameter, 1.8 µm of particles size (Agilent) using a binary gradient with formic acid 0.1% in water (solvent A), methanol/acetonitrile/formic acid (50/50/0.1 vol). The solvent B gradient program was 5% initial, isocratic for 1 min, increased to 28% in 4 min, to 55% in 20 min, to 90% in 2 min, isocratic for 3 min, equilibration to the initial conditions for 5 min. The column temperature was set at constant temperature of 30 °C, and the mobile phase flow rate at 0.25 mL/min. The identification of compounds was performed by using a TSQ Quantum™ Access MAX Triple Quadrupole Mass Spectrometer equipped with a HESI interface. The MS conditions were capillary temperature 330 °C; source heater temperature 280 °C; nebulizer gas N2; sheath gas flow 35 psi; auxiliary gas flow 10 arbitrary units; capillary voltage − 2.8 kV. Data were acquired in negative ionization mode using a data-dependent method. The data-dependent settings were: Full scan from 250 to 850 m/z, activation level 500 counts, isolation width 1 Da, default charge state 2, collision induced dissociation energy (CID) 35 eV, collision gas pressure 1.5 mTorr of Argon bip. All data were acquired and processed using Xcalibur v.2 (Thermo Fischer Scientific). The identification of compounds was achieved by comparing λ_max_, [M-H]^-^ and MS/MS fragmentation patterns with literature data^51^. Quantitative data were estimated by comparing the area of syringic acid to the area of each compound in the UV chromatogram at 280 nm. Syringic acid was used as internal standard, and was added to the sample before the injection as methanolic solution at 70 µg/mL final concentration.

### Solid phase micro extraction (SPME) followed by gas chromatography mass spectrometry GC/MS analysis of volatiles metabolites

The extraction of volatiles compounds was obtained using the solid phase micro extraction technique with a three-phase fiber, Divinylbenzene/Carboxen/Polydimethylsiloxane (DVB/CAR/PDMS) 50/30 µm, 1 cm length (Supelco). Samples (10 mg of flowers and leaves or 100 µL of extracts) were placed in 20 mL dedicated SPME vials, then equilibrated at 50 °C per 2 min, and finally the fiber was exposed in the headspace of vials for 5 min for volatiles absorption. The volatiles were desorbed by exposing the fiber in the injector port of the GC system heated at 230 °C. All the process of equilibration, extraction and injection was performed by robotic autosampler Combi-PAL tx. The Gas Chromatography coupled with Mass Spectrometry (GC–MS) was composed of a Clarus 680 GC equipped with an Elite-5 MS fused silica capillary column (30 m × 0.25 mm and 0.25 μm film thickness) and interfaced with a single quadrupole mass spectrometer Clarus SQ8C (Perkin Elmer). Mass spectra of target compounds were obtained by electron impact ionization system with standardized ionization energy of 70 eV. Helium 5.5 was used as a carrier gas at a constant flow rate of 1 mL/min. The injection was performed in splitless (closed split valve for 1 min) at 230 °C. The oven temperature was programmed from 50 °C to 110 °C at 3 °C/min, then raised to 230 °C at 5 °C/min, hold at the final temperature for 3 min. Transfer line and source temperatures were set at 250 °C. Data were collected in full scan mode in the range 33 − 300 m/z. Qualitative results include compound identification and area percentage of related peak in the total ions chromatogram. Compounds identification was performed by both Retention Indexes (RI) and mass spectra (MS) search in NIST and Wiley databases and bibliography^52^. The linear retention index of each identified compounds was calculated according to Van Den Dool and Kratz^53^. The reference standard for linear retention index calculation was the alkane standard mix C8-C20 (Supelco), it was injected using a 1:100 split ration and analysed with the upper mentioned chromatographic and mass spectrometric conditions.

## Results

### Vegetation and ecological characterization

The context in which *L. cuminoides* grows, in relation to its limited national and regional distribution, seems to be linked to microenvironments with low anthropic input. They are transitional environments between the natural habitats with mosaic of scrublands and annual meadows and cultivated fields with extensively management. The phytosociological survey showed that this taxon, in relation to its peculiar ecology, is one of the characteristic species of *Stipion retortae* O. de Bolòs 1957 (Syn.: *Stipion capensis* O. de Bolòs 1957) alliance, that encloses the plant communities of Western Mediterranean ephemeral winter pastures on loamy soils and over calcareous substrates^48^.

The vegetation was surveyed in two localities in the municipality of Statte, Province of Taranto (southern Italy) (relevés codes: 17-05-22-01 and 22-05-22-01). The sites have the same altitude (272 m a.s.l.), exposition (SW), slope (2°) and rockiness (2%), while the stoniness at Pineta di San Giovanni (PSG) site (30%) exceed of 10% that of the Gravina di Mazzaracchio (GM) (20%). The soil is always silty-loam, characterized by poor total carbonate, and low phosphorus availability but rich in organic carbon and total nitrogen. Remarkably, a significant higher number of individuals in the *L. cuminoides* population in the GM site was found, in addition to an increase of total coverage (Table 1).

**Table 1:** Phytosociological data related to the two sampling areas.

Locality	PSG	GM
Relevé code	17-05-22-01	22-05-22-01
Latitude (WGS84)	40°33.119′ N	40°32.193′ N
Longitude (WGS84)	17°10.143′ E	17°14.488′ E
Altitude (m.a.s.l.)	272	272
Aspect	W-SW	W-SW
Slope (°)	2	2
Relevé area (m^2^)	4	2
Stoniness (%)	30	20
Rockiness (%)	2	2
Cover total (%)	60	70
Average height of herbaceous layer (cm)	15	12
Individuals in population of *Lagoecia cuminoides* (estimate)	70	400
Habitat Directive 92/43 EEC	6220*	6220*
**Charact. ***Stipion retortae* O. de Bolòs 1957		
*Lagoecia cuminoides* L	1	3
*Ononis ornithopodioides* L	+	−
**Charact.** *Brachypodietalia distachyae* Rivas-Martínez 1978 and *Stipo-Trachynietea distachyae* S. Brullo et al. 2001		
*Trifolium scabrum* L	1	2
*Linum strictum* L. ssp. *strictum*	2	+
*Triticum biunciale* (Vis.) K.Rich	1	+
*Crupina crupinastrum* (Moris) Vis	+	1
*Hypochaeris achyrophorus* L	+	1
*Ononis reclinata* L	+	+
*Xeranthemum inapertum* (L.) Mill	+	+
*Valantia muralis* L	+	+
*Stipellula capensis* (Thunb.) Röser & H.R.Hamasha	1	−
*Stachys romana* (L.) E.H.L.Krause	−	1
**Transg.** *Helianthemetea guttati* Rivas Goday et Rivas-Mart. 1963		
*Trifolium stellatum* L	1	1
*Onobrychis caput-galli* (L.) Lam	1	+
*Trifolium campestre* Schreber	+	1
*Briza maxima* L	+	−
*Plantago bellardii* All. subsp. *bellardii*	−	** + **
*Helianthemum salicifolium* (L.) Miller	−	** + **
*Medicago minima* L	−	** + **
*Hedypnois rhagadioloides* (L.) F.W.Schmidt	−	** + **
**Other species**		
*Triticum vagans* (Jord. & Fourr.) Greuter	2	+
*Allium subhirsutum* L. subsp. *subhirsutum*	1	1
*Bellardia trixago* (L.) All	+	+
*Catapodium rigidum* (L.) Hubbard	+	+
*Lysimachia arvensis* (L.) U.Manns & Anderb	+	+
*Daucus carota* L. subsp. *carota*	+	+
*Petrosedum ochroleucum* (Chaix) Niederle	2	−
*Anisantha madritensis* (L.) Nevski subsp. *madritensis*	1	−
*Knautia integrifolia* (L.) Bertol. subsp. *integrifolia*	1	−
*Nigella damascena* L	1	−
*Asparagus acutifolius* L	+	−
*Avena barbata* Potter	+	−
*Centaurium tenuiflorum* (Hoffmanns. & Link) Fritsch	+	−
*Charybdis pancration* (Steinh.) Speta	+	−
*Geranium purpureum* Vill	+	−
*Lathyrus cicera* L	+	−

### Assessment of the conservation status in Italy

According to the IUCN protocol^49^, the only criterion B was used for the assessment of the conservation status of *L. cuminoides* in Italy. With an AOO less than 10 Km^2^, an EOO less than 100 Km^2^, a single location (sensu IUCN), and a continuing decline projected in area of occupancy, extent and quality of habitat, number of mature individuals, the species is Critically Endangered in Italy: CR B1ab(ii,iii,v) + 2ab(ii,iii,v).

### Antioxidant activity and total polyphenol content

Total polyphenols and antioxidant activity assays were performed on the ethanolic extract and in the water infusion extract. Results were calculated in mg per g of dry weight of plant material and shown in Table 2. The 2-ways ANOVA revealed no significant difference in total polyphenol content by comparing the sampling areas or the type of extraction. Nevertheless, the ethanol extracts of this taxon had two times higher antioxidant activity than the water infusion extract (p < 0.01). Comparing the two areas of collection, considering both ethanol and water extracts, samples collected in the second area (GM) reported a higher value of antioxidant activity.

**Table 2:** Antioxidant activity in µmol/g and total polyphenol in mg/g on dry weight of the extracts obtained from *L. cuminoides* samples collected in the two target areas (PSG = Pineta di San Giovanni; GM = Gravina di Mazzaracchio).

Extraction	Area	Total polyphenols	Antioxidant activity
Ethanol	PSG	5.9	32.2
	GM	7.5	36.1
Water	PSG	3.0	13.5
	GM	6.2	25.1
Standard error of the mean		0.6	0.7
2 ways ANOVA significance			
Extraction		0.083	< 0.01
Area		0.064	< 0.01
Extraction*area interaction		0.459	< 0.01

**Figure 4:**
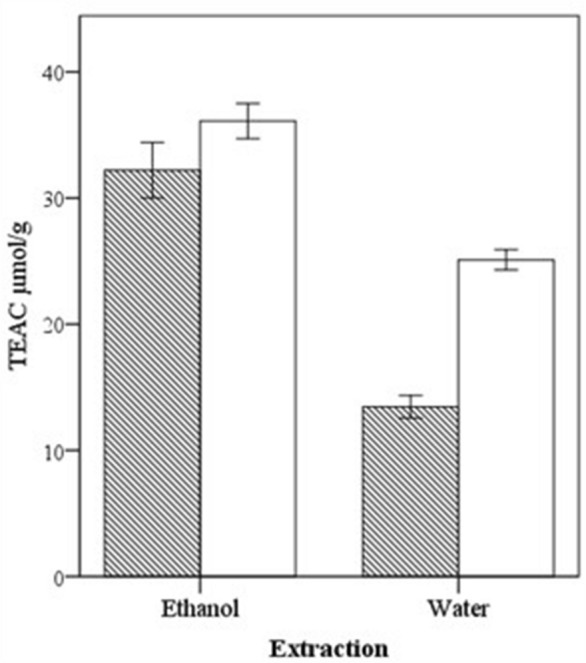
Results of the total antioxidant activity (TEAC) of *L. cuminoides* comparing the type of extraction and the site of collection (with pattern = PSG, white = GM).

As reported in Table 2, there was a significant interaction between the extraction method and the sampling area. This significance was illustrated in Fig. 4. In fact, the significant difference between the two areas could be highlighted only by the water infusion method.

It could be hypothesized that, in GM samples there were a higher concentration of some polar constituents with anti-scavenging activity than in the PSG samples, and that these compounds could be glycated flavonoids.

### Metabolites identification and quantification by chromatography techniques coupled to mass spectrometry (LC/MS–MS and GC/MS)

Non-volatiles metabolites present in the extracts obtained by water infusion or hydroalcoholic extraction were identified and tentatively quantified by ultra-high performance liquid chromatography coupled with triple quadrupoles detectors. Results show the presence of 12 compounds, ten of them were tentatively identified using literature data, whereas two unknown compounds were only described for their molecular mass, UV absorption and MS_2_ spectra (Table 3).

**Table 3:** Identification data obtained by LC/MS–MS of metabolites extracted from *L. cuminoides* samples.

Compound	RT	[M−H]^-^	Ms_2_	λ_max_ nm
3-Caffeoylquinic acid	7.28	353	191(100)	290, 330
Quercetin deriv.1	8.69	543	301(100)	272, 360
Quercetin pentosyl hex.1	9.2	595	301(100), 271(15)	256, 270, 356
Quercetin pentosyl hex.2	9.49	594	301(100), 271(15)	268, 360
Quercetin rutinoside	10.13	609	301(100), 271(20)	256, 270, 358
Quercetin 3-O-Gluc	10.34	463	301(100), 271(70)	256, 356
Quercetin deriv.2	11.27	433	271(100), 300(90), 255(20)	268, 360
Quercetin deriv.3	11.84	432	300(100), 151(60), 254(40)	256, 268, 356
Quercetin deriv.4	13.61	475	300(100), 271(40)	268, 356
Luteolin	16.72	285	134(100), 105(20)	270, 358
Unknown1	20.32	482	135(100)	294, 320
Unknown2	23.62	466	135(100)	294, 320

RT: retention time; [M−H]^−^: negative charged molecular mass m/*z*; Ms_2_: m/*z* signals pattern, relative intensity in brackets, obtained by collision induced dissociation from the [M−H]^−^ parent; λ_max_ nm: wavelength of maximum absorbance at peak apex.

Eight compounds were identified as quercetin derivate linked with one or more molecules of sugar and or organic acid, accordingly to Abas et al.^51^. In this case, the identification was based on the presence in MS_2_ spectrum of the characteristic signal at 301 m/*z* and the peak absorbance in the UV at 250 and 350 nm. The firs eluted compound was the caffeoylquinic acid, also known as chlorogenic acid, whereas the last ones are luteolin and the two unknowns.

In Table 4 the semiquantitative data of identified compounds are listed, comparing the hydroalcoholic and the hot water infusion extracts. The first five compounds, which are more hydrophilic were more concentrated in the water extract than in the ethanolic one. The opposite occurred for the last compounds, which are more hydrophobic.

**Table 4:** Semiquantitative data obtained by LC/MS–MS, expressed in µg/mL, of metabolites identified in the *L. cuminoides* samples extracts.

Compound	Ethanol extr	Water extr	Sign
3-Caffeoylquinic acid	7.7	13.7	**
Quercetin deriv.1	2.0	3.1	*
Quercetin pentosyl hex.1	3.7	6.1	*
Quercetin pentosyl hex.2	0.8	1.1	
Quercetin rutinoside	2.9	1.7	*
Quercetin 3-O-Gluc	15.4	9.0	*
Quercetin deriv.2	0.5	0.6	
Quercetin deriv.3	2.5	1.2	*
Quercetin deriv.4	1.6	2.2	
Luteolin	0.1	0.0	*
Unknown1	0.4	0.0	*
Unknown2	0.8	0.0	*

Ethanol: hydroalcoholic extract; water: extract obtained by hot water infusion; sign.: statistical significance obtained by ANOVA (*p < 0.05; **p < 0.01). The reported data are the mean value of all extracts obtained using the two compared methods, determined in duplicate.

A further analytical determination was performed to identify and compare the volatiles fraction of metabolites present in plant material and in the water and ethanolic extracts by SPME-GC/MS. The aim of this analysis is to evaluate the *L. cuminoides* as potential source of volatiles compounds with interesting biological activities in the plant aerial part and their fate during the preparation of a water or alcoholic extract for human consumption. The choice to use the SPME method of extraction instead of the classical hydrodistillation can be clarified taking into account the following considerations. First of all, *L. cuminoides* is a very rare species in the investigated area, it is a small plant and collecting all individuals found did not give a sufficient quantity for hydrodistillation nor to obtain a minimum quantity of essential oil to be quantified or analysed by GC/MS. Furthermore, the hydrodistillation produce an extract containing only part of the volatile compounds present in the matrix. The SPME is itself selective in relation to the extraction condition and the stationary phase used, but it is extremely more sensible then the hydrodistillation, and the chose to use a triple phase fibre gave us the possibility to have an exhaustive extraction, as much as possible, of the different chemical class of volatiles compounds.

Thirty-five volatiles’ compounds were identified in the head space of *L. cuminoides* aerial part of plant samples (Table 5). As expected, the elution order was monoterpens, oxygenated monoterpenes, sesquiterpene, and diterpenes. The most represented chemical group was that of sesquiterpenes, with 14 identified compounds. Not considering the quinone form of thymol, seven compounds were identified for both monoterpenes and oxygenated monoterpene groups. Four alcohols were detected, three C_6_ and one C_8_, and three of them had a double bond. Finally, one ester (3-octylacetate) and one diterpene (geranyl-p-cymene) were also detected.

**Table 5:** Results of volatiles metabolites identified in the plant and in the ethanol and water extracts of *L. cuminoides* by SPME-GC/MS.

Compound	RI	RI ref	Plant	Ethanol extr	Water extr	
**Alcohols**			**1.70**			
1-hexanol	863	865	0.32			
3-hexen-1-ol	849	851	0.69			
2-hexen-1-ol	860	857	0.66			
1-Octen-3-ol	977	976	0.04			
**Monoterpens**			**18.52**	**88.64**		
α-Thujene	923	924	0.07	0.59		
Sabinene	969	969	0.06			
α-myrcene	987	989	0.14	0.97		
α-Phellandrene	1004	1004	0.11			
α-Terpinene	1015	1017	0.22	0.38		
p-Cymene	1022	1024	3.19	18.55		
γ-Terpinene	1058	1054	14.73	68.15		
**Oxygenated monoterpens**			**21.26**	**8.72**		
Eucalyptol	1028	1031	0.47	1.86		
4-Thujanol cis-	1067	1066	0.10			
4-Thujanol trans-	1098	1098	0.26			
Terpinene-4-ol	1177	1177	0.86		2.23	
α-Terpineol	1193	1190	0.16			
Thymol	1290	1290	19.16	6.54	97.77	
Carvacrol	1293	1295	0.14			
Thymoquinone	1249	1249	0.10	0.32		
**Sesquiterpenes**			**58.37**	**2.63**		
α-Chamigrene	1412		0.12			
Caryophyllene (Z)	1417	1417	2.73	0.45		
Calarene	1421	1426	2.53			
γ-Elemene	1427	1431	0.49			
Aromadendrene	1436	1439	0.34			
β-Farnesene (E,E)	1458	1458	48.22	2.19		
α-farnesene (Z,E)	1490	1491	0.20			
Leden	1492	1493	0.28			
Bicyclogermacrene	1496	1493	0.46			
α-Farnesene (Z,Z)	1503	1507	0.44			
β-Bisabolene	1507	1507	0.19			
Selina-3,7(11)-diene	1536	1542	0.46			
Eudesma-3,7(11)-diene	1541	1545	0.56			
Germacrene B	1558	1558	1.35			
**Esters**						
3-octanyl acetate	1118	1117	0.03			
**Diterpenes**						
Geranyl-p-cymene	1950	1953	0.12			

Results are reported in area percentage of the total ions chromatogram. Compounds were listed following an increasing order of the linear retention index (RI); the bibliographic retention indexes (RI ref.) were also reported for comparison.

All identified compounds, apart from α-Chamigrene whose retention index was not found in bibliography, had a retention index close to the reference value plus or minus 6, validating in this way the identification process. Results in Table 5 show an important difference among the volatiles profile of plant and extracts. There is a considerable reduction of the number of volatile compounds present in the extracts in respect to the raw material. In fact, 10 compounds were identified in the head-space of hydroalcoholic extract. Considering the percentage of each chemical group, sesquiterpenes is the more affected by the extraction process, passing from 14 compounds and 58% of total area in the plat, to only 2 compounds and 2.63% of total area in the hydroalcoholic extract. Furthermore, sesquiterpenes are not detected at all in the water extract.

## Discussion

### Environmental and vegetation issue

**Figure 5:**
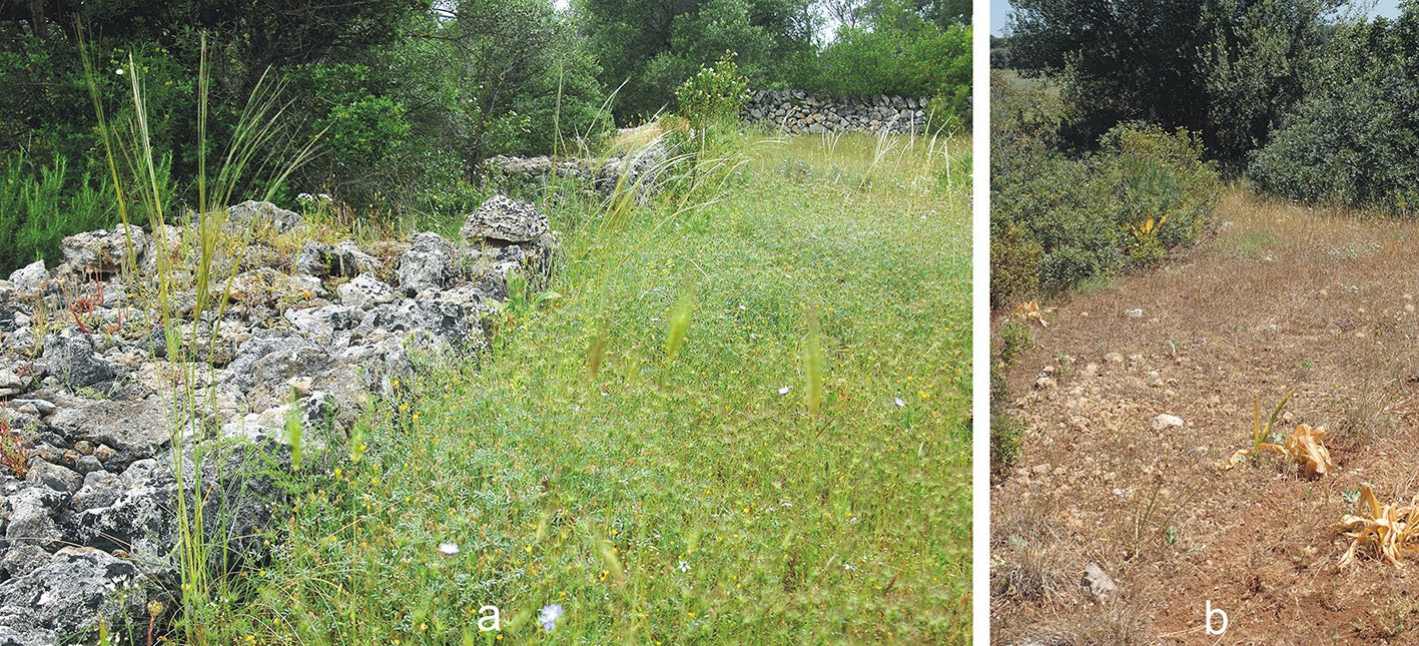
Plant communities on survey days. Pineta di San Giovanni (PSG) with *L. cuminoides* in bloom (**a**) (15 May 2023) and Gravina di Mazzaracchio (GM) (**b**) (20 May 2022). Pictures by E.V. Perrino.

The two studied sites have similar pedo-climatic characteristics, in fact they are geographically close one to each other and share climatic condition, altitude, vegetation cover, stoniness, rockiness and other vegetational features. The microenvironments in which *L. cuminoides* was detected, can be referred to the same annual meadows of *Stipion retortae* O. de Bolòs 1957 (syn.: *Stipion capensis* Br.-Bl. et O. de Bolòs ex Izco 1974) (Fig. 5). However, the vegetational context needs to be further investigated to define the *syntaxon* at the association level, including surveys in the other Mediterranean countries where it is already reported. From a conservation and management point of view the community aspects of this alliance fall within the priority habitat of the directive 92/43/EEC “Pseudo-steppe with grasses and annuals of the *Thero-Brachypodietea*” (code 6220*, the symbol * means that it is a priority habitat for the purposes of Directive 92/43/EEC), as the technical report in Subtype 3 (*Brachypodietalia distachyi*) includes many annual aspects, like *Stipion capensis* which is synonymous of *Stipion retortae*^54^.

The greatest threats to this habitat are the abandonment of traditional activities being integrated into so called cultural landscapes, never constituting the potential vegetation of their area. The abandonment of those activities thus triggers the reactivation of natural succession and therefore the substitution of those communities by others. When grazing disappears from these plant communities, the first effect is an increase in the cover of perennial species and decrease in biological diversity, and the consequence is the encroachment of woody vegetation as a result of the re-activation of natural succession. This situation results not only in the reduction in biodiversity but also in a dramatic increase in the risk of wildfire^55–57^. For these reasons the livestock is being increasingly used for creating and maintaining firebreaks in many Mediterranean countries^58, 59^. The abandonment of traditional activities usually results also in the disappearance of traditional infrastructures which might be important as observed in the first site with the dry-stone walls (Fig. 5a).

Another threat is the agricultural transformation taking away natural areas with results in an instantaneous disappearance of these plant communities. Especially due to their semi-pioneer character, it should be necessary a management, especially of grazing, to perpetuate them. Therefore, as a rule, traditional extensive management schemes should be considered as the desired conservation management model. The active management should be sheep or goat livestock, better if sheep, with a minimum impact of livestock on vegetation. The stocking rates must be never exceeding the capacity of 0.1 livestock unit ha^−1^ year^−1^^54^. The grazing system can be continuous with peaks in spring and sometimes autumn, depending on the time of onset of the autumn rain. Browse from shrub or forest formations and agricultural sub-products (e.g., stubble) are complementary sources of food for livestock. The traditional infrastructures, as dry-stone walls, play a key role in landscapes where these habitats, where they are usually of a high value for wildlife, and for the endangered plant species, as observed for *L. cuminoides*. Their conservation or restoration is therefore an advisable measure for this habitat type.

The environmental data collected and especially the differences in phenological stage between the two sites, with individuals in PSG in the flowering stage, those in GM with fully mature seeds, explain the slight differences in chemical composition and antioxidant activity in the Italian population of this taxon. Unfortunately, no scientific contribution gave useful elements on the aspects of environmental interaction and phenological stages on this taxon.

### Conservation status in Italy

*L. cuminoides* is a widespread species ranging from Portugal to Iran. However, in Italy it is very rare, with only two known sites, very close to each other, in Apulia region. Given to its restricted distribution and to the projected decline related to the detected threats, as the abandonment of pastoral traditional activities, the species is Critically endangered in Italy. Therefore, in situ and ex situ conservation actions should be implemented for the conservation of this rare and threatened species in Italy.

Interestingly, other species with a wide distribution range and very rare in Italy occur in Apulia, and they are also threatened with extinction in Italy^60–62^.

### Antioxidant activity and total polyphenol content

Ethanol and hot water infusion, as techniques for nutraceuticals compounds extraction, were chosen to investigate the potential use of this plant as infusion (like *Matricaria chamomilla*) or as ingredient in herbal mixture for liqueur production. In the supplementary materials (Table 1S) results of antioxidant activity and total polyphenols were reported in different units to make easier the comparison with already published results related to already known and used herbal species for these purposes. Considering the most commonly used plants worldwide to make hot water infusion preparation, we can consider *Matricaria chamomilla*, *Taraxacum officinale* (L.) W.W.Weber ex F.H.Wigg. and *Melissa officinalis* L. as references. Ivanova et al.^63^ investigated the polyphenols content and the antioxidant activity of the hot water infusion obtained using 26 Bulgarian plant species. The authors reported value range for total polyphenols (TP) 40–1700 µM (quercetin equivalent) and a total antioxidant activity ranging from 0.1 to 7 mM TEAC. The concentration of total polyphenols observed in the infusion of *L. cuminoides* expressed in quercetin equivalent ranged between 412 and 840 µM (Table 1S), whereas the antioxidant capacity was between 0.4 and 0.8 mM TEAC. These values are very close to that observed and reported for *M. chamomilla* and *Taraxacum officinale*. Similar conclusions could be deduced considering Jiménez-Zamora et al.^64^ who reported the antioxidant capacity and the total polyphenols content of 36 plant species commonly used for infusion. It was surprising to observe that the antioxidant activity of *L. cuminoides* (~ 1900 µmol/100 g as mean value) on dry weight has the same magnitude range of the green tea infusion (400–2000 µmol/100 g). Considering the results obtained on the hydroalcoholic extract, *L. cuminoides* infusion showed a TEAC of 34 µmol/g and a TP of about 6 mg of gallic acid equivalent (GAE) per gram dry weight^65^. Georgieva and Mihaylova^66^ reported a mean TEAC and TP for *M. chamomilla* ethanol extract of 0.45 µmol/g and 4.5 mg/g dw respectively. Finally, Issa-Issa et al.^67^ studied the TEAC and TP values of the ethanol extract of 15 plant species commonly used in the production of a Spanish liqueur, reporting 827 mg/L TEAC and 228 mg/L GAE for *M. chamomilla*. The values obtained in the present paper expressed in the same units of concentration were 285 mg/L TEAC and 225 mg/L GAE. All that considered, *L. cuminoides* showed interesting properties both if used as infusion or as source of antioxidants in the production of liqueurs.

### LC/MS–MS and GC/MS analysis

Since the TP and TEAC assays revealed interesting results on the extracts of *L. cuminoides* and values close to that of other species with well-known beneficial effects for human consumption, a deeply characterization of the obtained extracts was performed using the most advanced systems of LC/MS–MS for non-volatiles compounds, and the GC/MS for the volatiles ones.

As already mentioned in the results section, ten compounds were identified in the hydroalcoholic and in the water infusion extracts of *L. cuminoides* and most of them are glycated derivatives of quercetin flavonoid. Lin and Harnly^68^ studied the active substances extracted from *M. chamomilla* flowers during the hot water infusion preparation followed by LC–MS analysis. These authors identified 30 compounds among caffoyl quinic derivatives and glycated flavonoids. It is noteworthy that most of these later compounds have a common aglycone which is apigenin. In the extract of *L. cuminoides* both caffeoyl quinic isomers and glycated flavonoids were identified. These later showed a common aglycone which was quercetin. In both species lutein was also identified but for *L. cuminoides* only in the hydroalcoholic extract. There are many scientific reviews about the biological importance of quercetin and its derivates, among all these activities it should be highlighted the antioxidant, anticancer and antidiabetic effects on human health^69, 70^. By searching for the mean content of quercetin glucoside in the “phenols database”^71^ it was noted that quercetin 3 glucoside was found in a concentration range between 0.1 and more than 10 mg/100 mL considering wine and tea infusion among the different food sources of this flavonoid. Considering the quantitative data of the presented results obtained by LC–MS, the concentration of quercetin 3 glucoside in the water infusion or in the hydroalcoholic extract was 0.9 and 1.5 mg/100 mL respectively, within the upper mentioned range. Another important compound found in the extracts was the caffeoyl quinic acid. This compound is also known for its biological activity^72^ and the concentrations found (0.8 and 1.4 mg/100 mL in hydroalcoholic and in the water infusion extract respectively) are higher than that experienced in the black tea (0.3 mg/100 mL).

In our knowledge, only two papers discuss about the volatiles organic compounds of *L. cuminoides*. Bahmanzadagan et al.^73^ investigated the volatiles of *L. cuminoides* found in the south of Iran by hydrodistillation and head space analysis. These authors identified 35 volatiles, and among these, thymol, γ-terpinene and p-cymene were the most abundant. Baser and Tümen^28^ reported the volatiles composition of *L. cuminoides* collected in three different areas of Turkey and extracted by hydrodistillation. Also, these authors found thymol, γ-terpinene and p-cymene as the most abundant compounds. Apart from the differences in the percentages, probably related to the different techniques of extraction, both paper results are in agreement with the results of the present paper, highlighting a common biochemical pathway.

Considering the volatiles compounds identified in the plant material, β-farnesene, thymol, γ-terpinene and p-cymene were the most abundant. β-farnesene belongs to the sesquiterpene class. It is naturally produced by many plant species and by aphids as an alarm pheromone^74^. More recently, it is also studied as a potential anti-inflammatory modulator of human neutrophils^75^. Thymol, the second compound in percentage in Italian population of *L. cuminoides*, is a monoterpene widespread in many vegetable oils of wild plants^76^, especially in oil of *Thymus* sp. pl. Thymol is known as disinfectant in traditional medicine^77^, and antifungal^78^. It has also application in food packaging to prevent food spoilage during storage and increase the shelf life^79^. γ-Terpinene is another monoterpene present with a good frequency in this taxon, the third after thymol. The percentage found for γ-Terpinene (14.7%) in the analysed samples of *L. cuminoides* collected in the present work is close to the percentage value observed in the Iranian population of this species (15.6%)^73^, and in the Turkish populations (15.8%)^28^. The antibacterial and antifungal activity of γ-terpinene were documented by Yoshitomi et al.^80^, and Tahvilian et al.^81^ respectively. Finally, p-Cymene is a monoterpene found in over 100 plant species used for medicine and food purposes^82^. It shows a range of biological activities including antioxidant, anti-inflammatory, antinociceptive, anxiolytic, anticancer and antimicrobial effects^83, 84^. Considering the fate of these compounds during the preparation of the extracts, it worth to be mentioned the presence of all these four compounds in the headspace of the ethanolic extract but with an important increase of the relative percentage of γ-terpinene and p-cymene in respect to the value found in the plant material. Only two compounds, thymol and terpinene-4-ol, were detected in the hot water infusion, showing the low efficiency of water in extracting volatile terpenoids from vegetable sources.

## Conclusions

The present research on *Lagoecia cuminoides* allowed to: 1) characterize the vegetation of this very rare taxon, at least in Italy, with identification of the alliance (*Stipion retortae*) that falls within the priority habitat of the directive 92/43 EEC “Pseudo-steppe with grasses and annuals of the *Thero-Brachypodietea*” (code 6220*). This is a very important aspect for conservation purposes, suggesting to define its level of threat according to the IUCN guidelines; 2) assess the conservation status of *L. cuminoides* in Italy according to the IUCN guidelines; 3) deepen and clarify its metabolites with modern methodologies compared to the few previous works on this species; 4) evaluate the potential biological activity of the extracts obtainable by hot water infusion or hydroalcoholic extraction as preparation techniques for human consumption (decoction or liqueurs respectively). The results revealed a richness of *L. cuminoides* in beneficial compounds for human health (antioxidant, anti-inflammatory, antinociceptive, anxiolytic, and anticancer active compounds). It would be appropriate a collaboration with chemists and botanists in the other Mediterranean countries in which this species grows, to better define its phenotype and biological diversity in relation to the environmental factors, as well as evaluating its use in specific environmental restoration programs at Mediterranean level, as in REACT4MED (https://react4med.eu/) and EcoplantMed (http://www.ecoplantmed.eu/project/) projects.

The valorisation of *L. cuminoides* and its potential use in food and agronomical sectors could play an important role in any future development programs and strategies that aim to enhance the territory and foster the resilience of communities and natural habitats, especially in marginal areas, not suitable for agricultural purposes but extremely important for biodiversity safeguard.

### Supplementary Information

Supplementary Table 1.

## Data Availability

All data generated or analysed during this study are included in this published article and its supplementary information files.

## References

[1] Urbano M, Tomaselli V, Bisignano V, Veronico G, Hammer K, Laghetti G (2017). *Salicornia **patula* Duval-Jouve: From gathering of wild plants to some attempts of cultivation in Apulia region (southern Italy). Genet. Resour. Crop Evol..

[2] Accogli R (2023). Edible halophytes and halo-tolerant species in Apulia Region (Southeastern Italy): Biogeography, traditional food use and potential sustainable crops. Plants.

[3] Van Elsen T (2000). Species diversity as a task for organic agriculture in Europe. Agric. Ecosyst. Environ..

[4] Casella F, Vurro M, Valerio F, Perrino EV, Mezzapesa GN, Boari A (2023). Phytotoxic effects of essential oils from six Lamiaceae species. Agronomy.

[5] Perrino EV, Valerio F, Jallali S, Trani A, Mezzapesa GN (2021). Ecological and biological properties of *Satureja*
*cuneifolia* Ten. and *Thymus*
*spinulosus* Ten.: Two wild officinal species of conservation concern in Apulia (Italy). A preliminary survey. Plants.

[6] Valerio F, Mezzapesa GN, Ghannouchi A, Mondelli D, Logrieco AF, Perrino EV (2021). Characterization and antimicrobial properties of essential oils from four wild taxa of Lamiaceae family growing in Apulia. Agronomy.

[7] Linnaeus, C. *Species Plantarum* 1. Holmiae (1753).

[8] IPNI (The International Plant Names Index and World Checklist of Vascular Plants). http://www.ipni.org (2023). [accessed 15 February 2023]

[9] Barina Z, Pifkó D, Mesterházy A (2009). Contributions to the flora of Albania. Inventoring plant diversity of the Mediterranean. Willdenowia.

[10] Vangjeli, J. *Flora Albania Atlas* 1, 933 ( Koeltz Botanical Books Wilhelm Engelmann, 2017).

[11] Matevski, V. *et al*. *Flora and vegetation of the Macedonian steppe*. Ljubljana: Založba ZRC, ZRC SAZU, Biološki inštitut Jovana Hadžija ISBN 978-961-254-105-7 (2008).

[12] Seregin A (2008). Contribution to the vascular flora of the Sevastopol area (the Crimea): A checklist and new records. Fl. Medit..

[13] Mozaffarian, V. *Identification of Medicinal and Aromatic Plants of Iran, *1444 (Farhang Moaser, 2013).

[14] Bahmanzadegan A, Rowshan V, Zareiyan F, Hatami A (2019). *Lagoecia*
*cuminoides* L., its antioxidant activity and polyphenolic constituents from Iran. Nat. Prod. Res..

[15] Blakelock RA (1948). The Rustam Herbarium, Iraq, Part 1. Systematic List. Kew Bull..

[16] Ghazanfar SA, Edmondson JR (2013). Flora of Iraq.

[17] Euro+Med. Euro+Med PlantBase. The information resource for Euro-Med plant diversity (2023). https://ww2.bgbm.org/EuroPlusMed/ [accessed 15 February 2023].

[18] Tita, A. *Catalogus plantarum Horti Mauroceni*. Accedit Iter per Alpes Tridentinas. Patavii (1713).

[19] Saccardo, P.A. *Cronologia della Flora Italiana*: 177. Padova (1909).

[20] Bonato, G. A. *Catalogus plantarum Horti botanico-medici caes. reg. Academiae Patavinae.* Padova (1801).

[21] Cesati, V., Passerini, G., Gibelli, G. *Compendio della Flora Italiana*. F. Vallardi: Milano (1884).

[22] Groves E (1887). Flora della Costa Meridionale della Terra d’Otranto. N. Giorn. Bot. Ital..

[23] Caruel, T. *Flora Italiana* 8. Le Monnier: Firenze (1889).

[24] Fiori, A. *Nuova Flora analitica d’Italia* 2 (Firenze, 1925).

[25] Lattanzi E (2017). *Lagoecia*
*cuminoides* (Apiaceae): Una specie da ricercare nel Salento. Notiziario della Società Botanica Italiana.

[26] Silletti GN, Buono V (2022). Noterella 0367: *Lagoecia*
*cuminoides* L. Acta Plantarum Notes.

[27] Mattioli, P. A. *Commentarii in sex libros Pedacii Dioscoridis Anazarbei, De Medica materia, Liber tertium*: 579. Venetiis (1565).

[28] Baser HKC, Tümen G (1994). Composition of the essential oil of *Lagoecia*
*cuminoides* L. from Turkey. J. Essential Oil Res..

[29] Kunkel, G. *Plants for human consumption* (Koeltz Scientific Books, 1984).

[30] Holtom J, Hylton W (1979). Complete Guide to Herbs.

[31] Phillips R, Foy N (1990). The Random House Book of Herbs.

[32] Farida SHM, Ghorbani A, Ajani Y, Sadr M, Mozaffarian V (2018). Ethnobotanical applications and their correspondence with phylogeny in Apiaceae-Apioideae. Res. J. Pharmacogn..

[33] Usher G (1974). A Dictionary of Plants Used by Man.

[34] Linnaeus, C. *Philosophia botanica* 3 (Impensis Christiani Friderici Himburgi, 1790).

[35] POWO. *Plants of the World Online*. Facilitated by the Royal Botanic Gardens. http://www.plantsoftheworldonline.org/ (2023).

[36] Valiejo-Roman CM, Terentieva EI, Samigullin TG, Pimenov MG (2002). Relationships among genera in Saniculoideae and selected Apioideae (Umbelliferae) inferred from nrITS sequences. Taxon.

[37] Drude O, Engler A, Prantl K (1898). Umbelliferae. Die natürlichen Pflanzenfamilien.

[38] Wolff, H. In *Umbelliferae-Saniculoideae* (ed. Engler, A.) Das Pflanzenreich IV, 228 (Hf. 61), 1–305 (1913).

[39] Calestani V (1905). Contributo alla sistematica delle Ombrellifere d’Europa. Webbia.

[40] Cerceau-Larrival, M. T. Plantules et pollens d’Umbellifères. Leur intérêt systématique et phylogénatique. *Mémoires du Muséum national d'Histoire naturelle Sér. B ***14**, 1–16 (1962).

[41] Doğru-Koca A, Bagheri A, Moradi A (2020). Investigations on the phylogenetic position of the ditypic genus *Froriepia* reveal *Yildirimlia*, a new genus of Apiaceae. Taxon.

[42] Darlintong CD, Wylie AP (1956). Chromosome Atlas of Flowering Plants.

[43] Tutin, T. G. *et al*. *Flora Europaea* 1–5, 1st ed (Cambridge University Press, 1968–1980).

[44] Pignatti, S., Guarino, R., La Rosa, M. *Flora d’Italia* 1–4 (Edagricole, 2017–2019).

[45] Braun-Blanquet J (1964). Pflanzensoziologie Grundzüge der Vegetationskunde.

[46] Bartolucci F (2018). An updated checklist of the vascular flora native to Italy. Plant Biosyst..

[47] Galasso G (2018). An updated checklist of the vascular flora alien to Italy. Plant Biosyst..

[48] Mucina L (2016). Vegetation of Europe: Hierarchical floristic classification system of vascular plant, bryophyte, lichen, and algal communities. Appl. Veg. Sci..

[49] IUCN. *Guidelines for Using the IUCN Red List Categories and Criteria*. Version 15.1. Prepared by the Standards and Petitions Committee. https://www.iucnredlist.org/documents/RedListGuidelines.pdf (2022). [accessed 23 June 2023]

[50] Wrolstad RE (2005). Handbook of Food Analytical Chemistry.

[51] Abas F, Khatib A, Shaari K, Lajis NH (2014). Chemical characterization and antioxidant activity of three medicinal Apiaceae species. Ind. Crops Prod..

[52] Babushok VI, Linstrom PJ, Zenkevich IG (2011). Retention indices for frequently reported compounds of plant essential oils. J. Phys. Chem. Ref. Data.

[53] Van Den Dool H, Kratz PD (1963). Angeneralization of the retention index system including linear temperature programmed gas-liquid partition chromatography. J. Chromatogr. A.

[54] San Miguel, A. *Management of Natura 2000 habitats. 6220* Pseudo-steppe with grasses and annuals of the Thero-Brachypodietea* (European Commission, 2008).

[55] Stephens SL (2021). Fire, water, and biodiversity in the Sierra Nevada: A possible triple win. Environ. Res. Commun..

[56] Schlickman E, Milligan B (2022). Shepherding for wildfire adaptation: A case study of two grazing management techniques in the Mediterranean basin. Landsc. Architect. Front..

[57] Walesiak M, Mikusiński G, Borowski Z, Żmihorski M (2022). Large fire initially reduces bird diversity in Poland’s largest wetland biodiversity hotspot. Biodivers. Conserv..

[58] Pareja J, Baraza E, Ibáñez M, Domenech O, Bartolomé J (2020). The role of feral goats in maintaining firebreaks by using attractants. Sustainability.

[59] Celaya R (2022). Livestock management for the delivery of ecosystem services in fire-prone shrublands of Atlantic Iberia. Sustainability.

[60] Wagensommer, R. P., Fröhlich, T. & Fröhlich, M. First record of the southeast European species *Cerinthe retorta* Sibth. & Sm. (Boraginaceae) in Italy and considerations on its distribution and conservation status. *Acta Bot. Gallica Bot. Lett.* **161**(2), 111–115. 10.1080/12538078.2014.892438 (2014).

[61] Wagensommer, R. P., Perrino, E. V. & Silletti, G. N. *Carex phyllostachys* C.A. Mey (Cyperaceae) new for Italy and phytogeographical considerations. *Phyton ***54**(2), 215–222. 10.12905/0380.phyton54(2)2014-0215 (2014).

[62] Wagensommer, R. P. *et al.* First record for the flora of Italy and lectotypification of the name *Linum elegans* (Linaceae). *Phytotaxa* **296**(2), 161–170. 10.11646/phytotaxa.296.2.5 (2017)

[63] Ivanova D, Gerova D, Chervenkov T, Yankova T (2005). Polyphenols and antioxidant capacity of Bulgarian medicinal plants. J. Ethnopharmacol..

[64] Jiménez-Zamora A, Delgado-Andrade C, Rufián-Henares JA (2016). Antioxidant capacity, total phenols and color profile during the storage of selected plants used for infusion. Food Chem..

[65] Samaniego-Sánchez C (2011). The influence of domestic culinary processes on the Trolox Equivalent Antioxidant Capacity of green tea infusions. J. Food Compos. Anal..

[66] Georgieva L, Mihaylova D (2015). Screening of total phenolic content and radical scavenging capacity of Bulgarian plant species. Int. Food Res. J..

[67] Issa-Issa H (2019). Effect of the herbs used in the formulation of a Spanish herb liqueur, Herbero de la Sierra de Mariola, on its chemical and functional compositions and antioxidant and antimicrobial activities. Eur. Food Res. Technol..

[68] Lin LZ, Harnly JM (2012). LC-PDA-ESI/MS identification of the phenolic components of three Compositae spices: Chamomile, Tarragon, and Mexican arnica. Nat. Prod. Commun..

[69] Anand David AV, Arulmoli R, Parasuraman S (2016). Overviews of biological importance of quercetin: A bioactive flavonoid. Pharmacogn. Rev..

[70] Salehi B (2020). Therapeutic potential of quercetin: New insights and perspectives for human health. ACS Omega.

[71] Neveu, V. *et al.* Phenol-Explorer: An online comprehensive database on polyphenol contents in foods. *Database (Oxford)* **2010**, bap024. Doi: 10.1093/DATABASE/BAP024 (2010). 10.1093/database/bap024 PMC2860900 20428313

[72] Alcázar Magaña A (2021). Caffeoylquinic acids: Chemistry, biosynthesis, occurrence, analytical challenges, and bioactivity. Plant. J..

[73] Bahmanzadagan A, Hatami A, Rowshan V, Izadi M (2022). Chemical composition of essential oils using hydrodistillation and headspace methods of *Lagoecia*
*cuminoides*. Chem. Nat. Compd..

[74] Bayendi Loudit SM, Boullis A, Verheggen F, Francis F (2018). Identification of the alarm pheromone of cowpea aphid, and comparison with two other Aphididae species. J. Insect Sci..

[75] Schepetkin IA (2022). Neutrophil immunomodulatory activity of farnesene, a component of *Artemisia dracunculus* essential oils. Pharmaceuticals (Basel).

[76] Keefover-Ring K (2022). The chemical biogeography of a widespread aromatic plant species shows both spatial and temporal variation. Ecol. Evol..

[77] Begrow F, Engelbertz J, Feistel B, Lehnfeld R, Bauer K, Verspohl EJ (2010). Impact of thymol in thyme extracts on their antispasmodic action and ciliary clearance. Planta Medica.

[78] Dorman HD, Deans SG (2000). Antimicrobial agents from plants: Antibacterial activity of plant volatile oils. J. Appl. Microbiol..

[79] Sivaram S, Somanathan H, Kumaresan SM, Muthuraman MS (2022). The beneficial role of plant based thymol in food packaging application: A comprehensive review. Appl. Food Res..

[80] Yoshitomi K, Taniguchi S, Tanaka K, Uji Y, Akimitsu K, Gomi K (2016). Rice terpene synthase 24 (OsTPS24) encodes a jasmonate-responsive monoterpene synthase that produces an antibacterial γ-terpinene against rice pathogen. J. Plant Physiol..

[81] Tahvilian R (2016). Ethnomedicinal plants: Study on antifungal activity of essential oil of *Pistacia **khinjuk* (combined with the dominance γ-Terpinene) against *Candida albicans*. Int. J. Pharm. Clin. Res..

[82] De Oliveira TM (2015). Evaluation of p-cymene, a natural antioxidant. Pharm. Biol..

[83] Becer E, Mutlu Altundag E, Baser KHC, Vatansever HS (2022). Cytotoxic activity and antioxidant effects of *Origanum **onites* essential oil and its two major contents, carvacrol and p-Cymene on human colorectal (HCT116) and hepatocelluler carcinoma (HepG2) cell lines. J. Essential Oil Res..

[84] Marchese A (2017). Update on monoterpenes as antimicrobial agents: A particular focus on p-cymene. Materials.

