# Supplementary material for: Synecology of Lagoecia cuminoides L. in Italy and evaluation of functional compounds presence in its water or hydroalcoholic extracts
Source: Sci Rep. 2023 Nov 27;13:20906. doi: 10.1038/s41598-023-48065-w (PMC10684886; doi:10.1038/s41598-023-48065-w)
Supplement: Supplementary file 1 — Supplementary Table 1. [file 41598_2023_48065_MOESM1_ESM.docx]

| **Total polyphenols** | | | | | | | **TEAC** | | | | | | | |
| --- | --- | --- | --- | --- | --- | --- | --- | --- | --- | --- | --- | --- | --- | --- |
|  |  | µM Quercetin eq. | µg/mL | µg/g | mg/g | mg/100g | nmol/mL | µM | mg/L | nmol/g | µmol/g | µmol/100g | mg/g Trolox | % Inibition |
| Ethanol | PSG | 1029 | 252 | 7547 | 7.5 | 755 | 1204 | 1.21 | 301 | 36102 | 36.1 | 3610 | 9.1 | 57.7 |
|  | GM | 810 | 198 | 5939 | 5.9 | 594 | 1073 | 1.08 | 268.5 | 32200 | 32.2 | 3220 | 8.1 | 51.1 |
| Water | PSG | 840 | 205 | 6156 | 6.2 | 616 | 837 | 0.84 | 209.5 | 25096 | 25.1 | 2510 | 6.3 | 39.4 |
|  | GM | 412 | 100 | 3014 | 3.0 | 301 | 449 | 0.45 | 112 | 13460 | 13.5 | 1346 | 3.4 | 19.9 |
| Stand. Err. Mean | | 86 | 21 | 636 | 0.6 | 64 | 23 | 0.02 | 5.75 | 695 | 0.7 | 70 | 0.2 | 1.2 |
